# In Vitro Regeneration of *Ludwigia octovalvis* via Indirect Organogenesis and Evaluation of Its Bioactive Properties

**DOI:** 10.3390/antiox15070810

**Published:** 2026-06-28

**Authors:** Stephany Abigail Tadeo-Cuenca, Silvia Marquina-Bahena, Gabriel Alfonso Gutiérrez-Rebolledo, María Crystal Columba-Palomares, Araceli Guerrero-Alonso, Valeri Domínguez-Villegas, Francisco Cruz-Sosa, Mariana Sánchez-Ramos

**Affiliations:** 1 Department of Biotechnology, Metropolitan Autonomous University-Iztapalapa Campus, Av. Ferrocarril de San Rafael Atlixco 186, Col. Leyes de Reforma 1ª. Sección, Alcaldía Iztapalapa, Mexico City 09310, Mexico; cbs2223801823@izt.uam.mx; 2 Chemical Research Center-IICBA, Autonomous University of the State of Morelos, Av. Universidad 1001, Chamilpa, Cuernavaca 62209, Morelos, Mexico; smarquina@uaem.mx (S.M.-B.); araceli.guerreroa@uaem.edu.mx (A.G.-A.); 3 Natural Products Toxicology Laboratory, Department of Pharmacy, National School of Biological Sciences, National Polytechnic Institute, Av. Wilfrido Massieu, Gustavo A. Madero, Ciudad de México 07738, Mexico; ggutierrezr@ipn.mx; 4 Faculty of Pharmacy, Autonomous University of the State of Morelos, Av. Universidad 1001, Col. Chamilpa, Cuernavaca 62209, Morelos, Mexico; cpmc_ff@uaem.mx; 5 Faculty of Chemical Sciences and Engineering, Autonomous University of the State of Morelos, Av. Universidad 1001, Col. Chamilpa, Cuernavaca 62209, Morelos, Mexico; valeri.dominguez@uaem.mx; 6 Laboratory Technician School, Autonomous University of the State of Morelos, Av. Universidad 1001, Col. Chamilpa, Cuernavaca 62209, Morelos, Mexico

**Keywords:** indirect organogenesis, anti-inflammatory, antibacterial, antioxidant, secondary metabolites

## Abstract

Due to *Ludwigia octovalvis’* aquatic habitat’s vulnerability to climate change, this study developed an in vitro regeneration system using indirect organogenesis to ensure sustainable production of biomass and secondary metabolites. Treatment T16 (0.1 mg/L BAP and 1.0 mg/L NAA) was identified as the optimal hormonal regimen for callus induction and shoot differentiation. Phytochemical analysis by GC-MS revealed that seedlings regenerated under treatment T16 exhibited a diverse profile of 18 phytoconstituents, enhancing the accumulation of phytosterols, terpenes, and tocopherols. In vitro biological evaluation demonstrated that T16 extract possesses significant antibacterial activity (MIC < 62.5 µg/mL) against methicillin-resistant *Staphylococcus aureus*, and moderate antioxidant capacity. T16 extract showed anti-inflammatory effects superior to indomethacin at a low quantity (0.5 mg/ear) in adult CD1 mice of both sexes. In conclusion, the indirect organogenesis of *L. octovalvis* not only conserves the species but also optimizes its pharmacological potential, consolidating it as an efficient biotechnological platform for the development of advanced phytopharmaceuticals.

## 1. Introduction

*Ludwigia octovalvis* is an aquatic herb of the Onagraceae family that is not native but grows in tropical regions. In Mexico, it grows in Morelos, Oaxaca, and Veracruz states. *L. octovalvis* is used as a medicinal plant to treat skin conditions such as infections, erysipelas, pimples, rashes, and scabies, and is also used as a diuretic [1]. In other countries, *L. octovalvis* is used as a traditional treatment for edema, nephritis, dysentery, hypertension, dermatitis, boils, ulcers, impetigo, nervous diseases, orchitis, leorrhoea, headache, and swollen glands. Its decoction is consumed as a health drink, and also to treat diarrhea and flatulence [2,3,4,5,6]. To treat skin infections, the leaves are heated and soaked in alcohol before being applied. Additionally, for scabies, an ointment is prepared by macerating the plant and mixing it with lard and sulfur [1].

Previous studies showed that this herb extract possesses anti-diabetic, immunosuppressive activity; cytotoxic activity against human KB oral epidermoid carcinoma cells and Ht29 colorectal carcinoma; anti-helicobacter pylori, antimicrobial against *Staphylococcus aureus*, *S. epidermis*, *Candida albicans*, *C. krusei*, and *C. tropicalis*; glucosidase and lipase inhibitory activity; it also has hepatoprotective effects and attenuates cognitive impairment [3,7,8,9,10,11]. In addition, methanol leaf extract inhibits *E. coli* O157:H7 and *E. coli* ATCC 25922; root extract inhibits *Pseudomonas* spp.; and chlorophyll from the extract has antiproliferative activity on 3T3-L1 adipocytes [3].

Notably, the ethanolic extract (70%), formulated in aqueous creams at 1%, 3%, and 10% has demonstrated wound-healing activity by promoting wound contraction, re-epithelialization, fibroblast infiltration, and collagen deposition at the injury site. Moreover, no toxic effects were observed in acute dermal toxicity assays [12].

The species has been reported to be non-toxic upon oral administration and, at high doses (800 mg/kg), to significantly reduce cholesterol levels [6].

This species contains mainly flavonoid chemicals, phenolics, saponins, steroids, and tannins [6]. Other secondary metabolites (SMs) such as oleanoic acid, ursolic acid, beta-amyrin palmitate, beta-amyrin acetate, flavone C-glycosides (orientin, isoorientin, vitexin, and isovitexin), and five different olean-type triterpenes have also been identified and isolated [2,7,10,13].

As previously mentioned, *L. octovalvis* is of interest for its diverse pharmacological activities, so its phytochemical complexity could serve as an alternative source for new phytopharmaceuticals. This is why in vitro culture is used to produce these compounds in a constant, controlled manner, independent of biotic and abiotic factors, thereby providing a renewable and sustainable source for their production [14,15,16]. Through in vitro culture techniques, rapid propagation can be achieved, yielding useful variants in medicinal species and conserving germplasm, which in turn offers an advantage for their production for pharmaceutical and commercial purposes [17,18].

One of the techniques used is the regeneration of seedlings through organogenesis, which can occur via two morphogenetic pathways: direct and indirect. In direct organogenesis, organs form from competent cells or tissues of the explant without an intermediate callus phase, whereas in indirect organogenesis, regeneration occurs via a previously induced callus, from which roots or shoots subsequently differentiate. The transition from cell proliferation to organogenic differentiation is primarily regulated by the interaction between auxins and cytokinins. In general terms, high levels of auxins or a high auxin-to-cytokinin ratio favor callus induction and proliferation and the formation of adventitious roots, while a decrease in this ratio, associated with greater availability of cytokinins, promotes the acquisition of stem identity and shoot regeneration. It is now recognized that this hormonal balance acts through complex signaling networks and gene regulation that control cell reprogramming, the acquisition of pluripotency, and the establishment of new meristems during plant regeneration [19,20,21].

Given the empirical therapeutic applications of *L. octovalvis* in traditional medicine across cultures, it is imperative to design sustainable preservation strategies that do not compromise wild populations. In this regard, in vitro regeneration offers a viable alternative for its conservation and use. However, a standardized and efficient for the production of biologically active compound in this species has yet to be fully investigated. That is why the objective of this study was to standardize a regeneration protocol using leaf and nodal explants and to evaluate the effects of different concentrations and combinations of auxins and cytokinins in a solid culture medium. Furthermore, the study aims to verify that organic extracts from regenerated plantlets retain the traditionally attributed biological properties of this species, thereby establishing a biotechnological platform for the sustainable, continuous production of phytocompounds with therapeutic potential.

## 2. Materials and Methods

### 2.1. Plant Material

Complete *L. octovalvis* plant was collected on 7 September 2022, in Lomas de Zomplantle, Morelos state, Mexico. Botanical identification of the species was carried out by Biol. Gabriel Flores Franco and deposited in the Herbarium of the Autonomous University of the State of Morelos (HUMO), Mexico, with folio number 39816.

### 2.2. Seed Disinfection

Seeds were placed in Whatman No. 1 filter paper packets (2 × 2 cm) for disinfection under aseptic conditions in a laminar flow hood. Process began with a 15-min wash in a 150 mL solution of sterile distilled water with added surfactant Axion® (Colgate-Palmolive, Mexico City, Mexico)under constant agitation. Subsequently, packets were immersed in a 1% (*v*/*v*) commercial sodium hypochlorite Cloralex® (Alen, Monterrey, Mexico) solution for 20 min. After disinfection, three successive rinses were performed with sterile distilled water (5 min each) under constant agitation. Finally, seeds were removed from packets into sterile Petri dishes and sown (5 seeds per packet) in culture tubes containing 15 mL of previously sterilized Murashige and Skoog (MS) medium [19].

### 2.3. Seeding in Culture Medium

After disinfection, seeds were inoculated into culture tubes (25 × 150 mm) containing 15 mL of MS medium, supplemented with sucrose (3%, *w*/*v*) and gelled with Phytagel^®^ (0.25%, *w*/*v*) [22,23]. The pH of the medium was adjusted to 5.7 ± 0.1 by adding NaOH (97%; TECSIQUIM, Toluca de Lerdo, Mexico) or HCl (38%; J.T. Baker, Phillipsburg, NJ, USA) (0.1 N) before autoclaving at 121 °C for 20 min. Cultures were incubated at 25 ± 2 °C under a 16 h photoperiod with a light intensity of 50 µmol m^−2^ s^−1^ provided by white and fluorescent lamps. During the initial phase, seeds were maintained under these conditions for 3 d, and subsequently, the resulting seedlings were maintained by biweekly subcultures in the same culture medium. These seedlings were used as the control group without growth regulators, designated “PW”.

### 2.4. Hormonal Treatments

The effect of various plant growth regulators (PGRs) on the morphogenic response of leaf and nodal explants (0.5–1.0 cm) from 30-day-old in vitro plantlets was evaluated. Explants were cultured in MS medium supplemented with different combinations of auxins and cytokinins: 2,4-Dichlorophenoxyacetic acid (2,4-D) (95%; Sigma-Aldrich, St. Louis, MO, USA) and naphthaleneacetic acid (NAA) (97%; Sigma-Aldrich, USA) were used as auxin sources, while 6-benzylaminopurine (BAP) (≥98%; Sigma-Aldrich, USA) and kinetin (KIN) (≥96%; Sigma-Aldrich, USA) were used as cytokinin, all at concentrations of 0.0, 0.1, 0.5, and 1.0 mg/L. Experimental design comprised 50 treatments (designated by the letter T), each with five replicates per explant type and four explants per flask, for a total of 20 experimental units per treatment.

### 2.5. Biomass Extraction and Phytochemical Profile by GC-MS

Cultures exhibiting a morphogenic response (direct and indirect organogenesis and callus induction) were propagated until a biomass sufficient to reach 5 g dry weight was obtained. The material was ground and subjected to sequential extraction by maceration at room temperature using solvents of increasing polarity (hexane, ethyl acetate, and methanol) in three 72 h stages. Resulting extracts were gravity filtered through Whatman No. 1 filter paper and concentrated to dryness (maximum 40 °C) by evaporation under reduced pressure in a digital rotary evaporator (model D-402-10; PRENDO, Puebla, Mexico).

The phytochemical profile of the extracts from the treatments (T6, T16, T35, T36, T37, and T40) and the control group PW was determined by gas chromatography-mass spectrometry (GC-MS). An Agilent 6890 system equipped with a 5973N mass-selective detector (Agilent Technologies, Santa Clara, CA, USA) was used. For the analysis, 1.0 mg aliquots of each extract were dissolved in 1 mL of HPLC-grade chloroform and immediately injected into the system. Analytical separations were performed on a nonpolar stationary-phase HP-5MS capillary column (30 m × 0.25 mm i.d., 0.25 µm film thickness). High-purity helium was used as the carrier gas at a constant flow rate of 1 mL/min. The oven temperature program began with an isothermal period at 40 °C for 1 min, followed by a linear temperature ramp to 250 °C. Ionization was performed by electron impact (EI) at 70 eV, with an electron multiplier voltage of 1859 V [24].

Structural elucidation of the metabolites was carried out by comparing their mass spectra with records from the NIST library (version 1.7a). An identity criterion was established based on a match score greater than 80%, supplemented by detailed analysis of characteristic fragmentation patterns. Quantification of the components was performed by integrating the areas under the peaks in the chromatograms [25], expressing the results as relative abundance (%).

### 2.6. Antibacterial Assay

#### 2.6.1. Microorganisms

Antibacterial activity of extracts derived from T16 treatment and control PW was evaluated in *Staphylococcus aureus* (ATCC 6538), *Methicillin-resistant Staphylococcus aureus* (ATCC 43300), *Streptococcus pyogenes* (ATCC 19615), *Escherichia coli* (ATCC 8739), *Salmonella typhimurium* (ATCC 14028), and *Pseudomonas aeruginosa* (clinical isolates). All microorganisms were incubated under aerobic conditions at 37 °C.

#### 2.6.2. Broth Dilution Method

Antibacterial susceptibility was determined by the broth microdilution method, following the guidelines of the Clinical and Laboratory Standards Institute (CLSI) M100 standard [26,27]. Extracts (T16 treatment and control PW) were evaluated at concentrations ranging from 31.25 to 500 µg/mL, after initial dissolution in 10% (*v*/*v*) dimethyl sulfoxide (DMSO) (≥99.9%; Sigma-Aldrich, USA) and subsequent dilution in Müeller–Hinton broth (Difco^TM^; Becton Dickinson, Franklin Lakes, NJ, USA). Gentamicin (Sigma-Aldrich, 3050 Spruce St. Louis, USA) was used as a positive control at concentrations of 0.15 to 40 µg/mL, while 10% (*v*/*v*) DMSO was used as a negative control.

Bacterial *inoculum* was prepared in saline solution (0.85%), adjusted to 0.5 McFarland standard, and then diluted to a final concentration of 5 × 10^5^ CFU/mL. Growth controls (100 µL of broth and 100 µL of *inoculum*) and sterility controls (200 µL of broth) were established on microtiter plates. For experimental samples, each well was inoculated with 100 µL of bacterial suspension and 100 µL of extract solution, and then the plates were incubated at 37 °C for 24 h. Finally, absorbance was measured at 600 nm using a Glomax detection system TM297 (Promega, Madison, WI, USA). The minimum concentration of antibacterial agent responsible for the inhibition of bacterial growth was defined as the minimum inhibitory concentration (MIC). After incubation, 10 µL of MTT (0.4 mg/mL) was added to each well, and the samples were incubated for 3 h at room temperature to determine the bactericidal or bacteriostatic effect.

### 2.7. In Vitro Antioxidant Capacity

The antioxidant activity of the methanolic extracts from the plant samples (T16 treatment and control PW) was evaluated using the ABTS and DPPH assays, total phenolic content analysis, and flavonoid determination.

#### 2.7.1. Total Phenolic Content

The TPC quantification was carried out by the Folin–Ciocalteu method [28]. Briefly, an aliquot of 28 μL of extracts dissolved in 90:10 H_2_O-MeOH (0.9 mg/mL) was mixed with 42 μL of Folin–Ciocalteu reagent (1 N). The mixture was kept on an orbital shaker at 80 ± 1 rpm and incubated at room temperature in the dark for 5 min. Then, 42 μL of a Na_2_CO_3_ solution (20%; *w*/*v*) was added and 168 μL of distilled water, incubated for 30 min under the same conditions. Samples were immediately read against a blank on a Multiscan Go spectrophotometer at 760 nm. The TPC in the samples was calculated by comparing the optical density (OD) of a gallic acid standard curve (5–100 μg/mL; R^2^ = 0.9998; y = 0.0097x + 0.0656). The results are expressed as mg of gallic acid equivalents per g of dry extract (mgGAE/g extract). All determinations were made in triplicate (*n* = 3) and are reported as the mean ± standard deviation.

#### 2.7.2. Determination of Flavonoid Content

Flavonoid content was determined using the aluminum chloride assay according to [29]. Briefly, an aliquot (28 μL) of the extract was mixed with 112 μL of distilled water, and 8.4 μL of 5% NaNO_2_ was added. After 5 min of incubation, 8.4 μL of 10% AlCl_3_ was added. After 1 min, 56 μL of 1 M NaOH was added, and the volume was adjusted to 280 μL with 67.2 μL of distilled water. After 10 min, the absorbance of the resulting solution was measured at 510 nm. Catechin was used as a standard curve (5–100 μg/mL; R^2^ = 1; y = 0.0024x + 0.046) to express total flavonoid contents of samples as mg catechin equivalent per g of sample (mgCAE/g sample). All the samples were analyzed in triplicate.

#### 2.7.3. DPPH Radical Scavenging Assay

The antioxidant activity of the extracts was also evaluated by the DPPH • method [30]. The radical DPPH (200 mM) was dissolved in methanol and incubated at room temperature, and in the dark for 30 min. Briefly, an aliquot of 50 μL of each extract (1–300 μg/mL) or gallic acid as a positive control (1–100 μg/mL) dissolved in methanol was mixed with 50 μL of DPPH • (200 mM).

The mixture was incubated at room temperature and in the dark for 30 min. Subsequently, the samples were read at 517 nm. The results were expressed as IC_50_ and were measured in triplicate (*n* = 3).

#### 2.7.4. ABTS•+ Cationic Radical Inhibition Assay

The ability to scavenge free radicals was determined by 2,2′-azino-bis(3-ethylbenzothiazoline-6-sulfonate) (≥98%; Sigma-Aldrich, USA), ABTS•+ cationic radical decolorization assay [31]. The ABTS•+ chromophore was generated by a direct oxidation reaction by mixing an ABTS solution (7 mM) with potassium persulfate (K_2_S_2_O_8_, 2.45 mM; ≥99.0%, Sigma-Aldrich, USA) in a stoichiometric ratio of 1:1. This mixture was kept in total darkness at room temperature (25 ± 2 °C) for 12–16 h to ensure radical formation and stabilization.

Prior to quantification, the stock solution was diluted in acetate buffer (pH 4.5) to a standard absorbance of 0.70 ± 0.02 at 734 nm. For the experimental assay, 10 µL of each extract (concentration range: 12.5 to 250 µg/mL) or Trolox standard (10 to 60 µM, used as a positive control; 97%, Sigma-Aldrich, USA) was combined with 200 µL of ABTS•+ solution in 96-well microplates. After the mechanical shaking and incubation in the dark for 7 min, the optical density was measured at 734 nm using a microplate reader (Agilent Technologies, USA).

### 2.8. In Vivo Evaluation

#### 2.8.1. Animals

Healthy adult 60 male and 60 female CD1 mice (20 ± 3 g) were obtained from Animal *Vivarium* de PROPECUA S.A. and kept in plastic cages for a 7-day conditioning period prior to the experiments under laboratory conditions (12 h/12 h light/dark cycles; temperature 25 ± 2 °C; humidity 55–80% and fed Rodent Chow and water *ad libitum)*. Experiments were conducted in accordance with the statutes of the International Committee for the Care and Use of Laboratory Animals (IACUC) and Mexican Official Norm (NOM-062-ZOO-1999, modified in 2001), revised in 2026. The experimental protocol was also reviewed and approved by the Bioethics Committee of the Autonomous University of the State of Mexico (UAEMex), with registration number 01-01-26.

#### 2.8.2. TPA-Induced Mouse Ear Edema

This preclinical model was evaluated following protocol previously described by Gutiérrez-Rebolledo et al. [32], being a preclinical model to understand the local/dermal anti-inflammatory potential of possible COX-2 inhibitory agents, similar to NSAIDs. Inflammation was induced with 2.5 µg/ear of 12-O-tetradecanoylphorbol-13-acetate (TPA) (2.5 µg) (≥99%; Sigma-Aldrich, USA), dissolved in 25 µL of acetone (≥99%, JT Baker, USA), applied to the inner and outer surfaces of the mice’s right ears (W_t_); 25 µL of just vehicle (acetone) were applied to the left ears of all animals as basal weight control (W_nt_).

Male and female mice were randomly distributed (*n* = 6) in each experimental group per sex: un-treated TPA control (I), indomethacin (Sigma-Aldrich, USA) (II–IV), ethyl acetate extracts of T16 treatment (V–VII), and ethyl acetate extracts from control PW (VIII–X). All experimental groups received TPA, and 30 min later only treated groups were skin applied with 0.5, 1, and 2 mg/ear. All samples from the different treatments were dissolved in 50 µL of acetone.

Six hours after application of TPA, animals were euthanized by cervical dislocation. Circular sections 6 mm in diameter were taken from the treated right ear (t) and the untreated (nt) left ear, which were weighed (mg) to determine inflammation.

Percentage of inhibition (%) was determined comparing the data of each treated group against the untreated TPA control using following formula:

% = ((Δ_W_ TPA control − Δ_W_ treatment)/Δ_W_ TPA control) × 100, (1)

where Δ_W_ = W_t_ − W_nt_, being Wt the weight of the section of the treated ear and Wnt the weight of the section of the non-treated ear of same mouse.

### 2.9. Statistical Analysis

Experiments were conducted using a completely randomized factorial design. Each treatment consisted of three independent replicates, using four explants per experimental unit. Response variables included callus induction percentage and morphogenic response frequency, reported as standard error (±) of the mean (SEM). A two-way analysis of variance (ANOVA) was performed to assess significance of main effects (PGR concentration and explant type) and their interaction on in vitro development. In cases where significant differences were detected, Tukey’s multiple comparison test was applied (*p* < 0.05). Data from in vitro antioxidant capacity assays was analyzed using one-way ANOVA followed by Tukey’s test. For in vivo anti-inflammatory activity assays, data was analyzed by two-way ANOVA followed by Student–Newman–Keuls (SNK) post hoc test. All statistical analyses were performed using GraphPad Prism (version 9.4.1, GraphPad Software, Inc.) (*p* < 0.05).

## 3. Results and Discussion

### 3.1. Induction Response

Regeneration of *L. octovalvis* seedlings was determined by concentration and combination of PGRs, with responses observed in both types of explants (Table 1). In contrast, 2,4-D, applied individually or in combination with BAP, did not induce seedling formation in either nodal or foliar explants, regardless of tested concentrations. This behavior is consistent with that reported in *Launea taraxacifolia* [33]; however, in other species, this auxin, alone or in combination with cytokinins, has been documented to promote somatic embryogenesis [34,35,36,37]. These differences in response could be associated with the state of auxin homeostasis, a key factor in embryonic development, which depends on hormonal balance influenced by species-specific endogenous load [38,39]. Furthermore, interaction between adequate concentrations of auxins and cytokinins is essential for the success of in vitro micropropagation in most plant species [40].

Combination of KIN and NAA significantly promoted regeneration in both types of explants, with statistically significant differences between treatments. Responses were observed in both leaf explants (T35, T39, and T40) and nodal explants (T32, T35, T36, T37, T38, and T39), with treatment T40 showing the highest regeneration rate (83.33%) (Table 1). Similar results have been reported in other studies, where interaction between these plant hormones favored seedling propagation from different types of explants [41,42,43,44].

Single application of phytohormones produced variable responses depending on PGR used and type of explant. KIN stimulated seedling regeneration in nodal explants, with values equal to or greater than 30%, and no significant differences between evaluated concentrations. This result is consistent with its well-documented role in inducing shoots and seedlings in various plant species. For example, 83% regeneration has been reported in *Cucumis sativus* seedlings with 1 mg/L of KIN [45], multiplication rates of 94.4% in *Quercus leucotrichophora* roots at the same concentration [46], as well as simultaneous induction of shoots and roots from *Matthiola incana* nodes using KIN concentrations between 0.5 and 2 mg/L [47]. In contrast, NAA induced regeneration only in nodal segments (25%) at a concentration of 0.5 mg/L (T8). At this same concentration, an increase in root and seedling formation with leaf primordia in banana has been reported [48], as well as maximum root induction in *Punica granatum* [49].

Only BAP application resulted in moderate regeneration (25%) in both explant types (T1 and T2). Similarly, its application at 0.5 mg/L increased shoot formation in nodal explants of *Aquilaria hirta* [50]; however, its combination with NAA showed an explant-type-dependent effect, significantly favoring regeneration in leaf tissues. Among tested treatments, T16 showed the highest regeneration rate (65%), suggesting that this hormonal interaction is more efficient in leaf explants, possibly due to differences in the physiological state and endogenous hormone content of each tissue type (Table 1). Consistently, combination of BAP and NAA has promoted shoot proliferation via indirect organogenesis in *Chenopodium quinoa* Willd. and in vitro regeneration in *Prunella vulgaris* [51,52].

Finally, combination of KIN and 2,4-D induced regeneration exclusively in leaf explants, with a rate of 33.33% (T48), confirming 2,4-D low efficacy promoting efficient morphogenetic responses in *L. octovalvis*, even when combined with cytokinins, as previously observed in other species [37].

Overall, the results indicate that organogenesis in *L. octovalvis* is strongly influenced by both the interaction between auxins and cytokinins and the type of explant used, reflecting a balance between endogenous plant tissue hormones and exogenous concentrations supplied in the culture medium. In this context, the present study contributes to defining an efficient hormonal scheme for in vitro regeneration of this species, providing a solid basis for future micropropagation and conservation work.

### 3.2. Shoot Organogenesis

**Figure 1:**
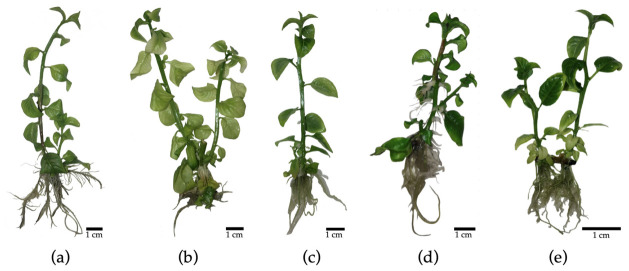
Shoots obtained through direct organogenesis from nodal segmentations. Treatments: (**a**) negative control without PGRs (PW); (**b**) T32 (0.1 mg/L KIN + 0.1 mg/L NAA); (**c**) T35 (0.1 mg/L KIN + 1 mg/L NAA); (**d**) T36 (0.5 mg/L KIN + 0.1 mg/L NAA); (**e**) T13 (1 mg/L KIN).

In treatments T13, T32, T35, and T36, variants regenerated through direct organogenesis were identified. These showed morphological differences compared to control seedlings PW (grown without PGRs), particularly in color, size, and thickness of both leaves and stems (Figure 1). Direct organogenesis was determined from macroscopic observations and morphological characterization, demonstrating the formation of shoots without a prior callus formation phase.

Treatments T32, T35, T36, and T13 showed less root formation compared to the control (Figure 1a). Treatment T32 (Figure 1b) resulted in seedlings with a greater number of light green leaves, while treatment T35 (Figure 1c) showed less leaf development. Treatment T36 (Figure 1d) showed adventitious root formation, a slightly more robust stem, and more intense green pigmentation compared to control plants. Treatment T13 (Figure 1e), on the other hand, induced the development of variegated leaves with light and dark green hues.

Observed morphogenetic variations could be attributed to the multifaceted functions of auxins in plant development, including leaf initiation, vascular differentiation, and root formation [53,54]. Similar results have been documented in other species, such as *Artemisia scoparia* and *Phalaenopsis Golden Peoker*, in which the combined application of KIN and NAA promoted direct organogenesis and induced morphological modifications in regenerated shoots [55,56].

**Figure 2:**
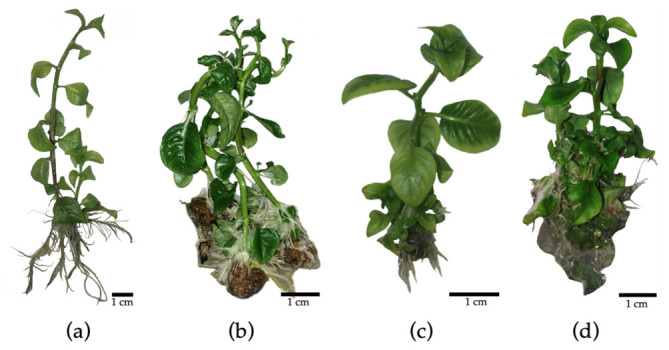
Shoots obtained through indirect organogenesis. Treatments and explant types: (**a**) negative control without PGRs (PW); (**b**) T16 (0.1 mg/L BAP + 1 mg/L NAA), leaf; (**c**) T37 (0.5 mg/L KIN + 0.5 mg/L NAA), node; (**d**) T40 (1 mg/L KIN + 0.5 mg/L NAA), leaf.

In addition, treatments T16, T37, and T40 induced regeneration via indirect organogenesis in both types of explants, characterized by initial callus formation on day 6, followed by shoot differentiation through day 40 (Figure 2). These observations demonstrate a significant influence of the hormonal composition of the medium on the regenerative pathway and the morphological characteristics of the seedlings obtained.

From a morphogenetic perspective, treatments T16, T37, and T40 produced seedlings with larger leaves and more intense color than the control plant (Figure 2a). Variants T37 and T40 (Figure 2c,d) had variegated leaves and more prominent callus formation than that observed in T16 (Figure 2b). Treatment T16 contained the highest concentration of auxin (1 mg/L NAA), which could explain the greater elongation observed, since auxins regulate stem elongation [57]. In contrast, T37 and T40 treatments shared the same concentration of NAA (0.5 mg/L), which could justify their morphological similarities; however, T40 had a higher concentration of cytokinin (1 mg/L KIN), which possibly contributed to the formation of more noticeable callus and more intense pigmentation, considering the role of cytokinin’s in cell division, chloroplast development, and bud differentiation [58].

### 3.3. Extract Obtention and Phytochemical Analysis

Using GC-MS, a total of 36 metabolites were identified in all extracts, including control without PGRs (Table 2). Among the most ubiquitous compounds detected in the samples were squalene, *α*-tocopherol, campesterol, stigmasterol, *γ*-sitosterol, *β*-sitosterol, lupeol, phytol, and dihydroxanthine. Persistent presence of these metabolites is consistent with previous reports in ethanolic extracts of wild specimens of *L. octovalvis* [59]. This agreement not only validates the phytochemical characterization of the biotechnological culture model but also confirms the stability of the species’ biosynthetic profile, even under in vitro culture conditions.

In the present study, *L. octovalvis* in culture medium supplemented with plant hormones favored biosynthesis of secondary metabolites (SMs) not previously reported in wild plants of this species. These include alcohols and terpenes such as 1-octanol, 2-butyl-, endo-borneol, *α*-terpineol, and trans-geranylgeraniol; fatty acids and derivatives such as *n*-hexadecanoic acid, (*Z*)-13-octadecenal, 13-tetradecenal, oleic acid, stearic acid, and dodecanoic acid, 3-hydroxy-; as well as high biological value SMs, including pterin-6-carboxylic acid, *α*-tocospiro A and B, *γ*-tocopherol, cucurbitacin B (25-deacetoxy-), olean-12-ene-3,28-diol (3β-), betulin, phytonadione, and the coumarin 7-hydroxy-3-(1,1-dimethylprop-2-enyl)coumarin (Table 2). This diversification of the chemical profile suggests that the composition of the culture medium and PGR concentrations act as chemical signals that activate or modulate latent biosynthetic pathways [60,61]. In addition to regulators, factors such as genotype, temperature, and photoperiod synergistically influence secondary metabolism, enabling the biosynthesis of SMs not typically expressed under wild conditions.

In contrast, seedlings grown in PGRs’ absence exhibited a more restricted phytochemical profile, with only 16 of the previous 36 identified compounds detected across all treatments in the PGRs-supplemented medium. This marked difference suggests that in vitro culture under controlled conditions, particularly when supplemented with phytohormones, stimulates the activation of specific biosynthetic pathways involved in SMs production.

These findings demonstrate the metabolic plasticity of *L. octovalvis* and are consistent with previous reports describing how hormonal stress and plant tissue culture conditions can induce the accumulation of particular phytochemicals that are not expressed, or are minimally expressed, under basal conditions [62,63].

Among PGRs-supplemented cells, treatments T16, T32, and T35 stood out for exhibiting the greatest phytochemical diversity, with a total of 18 identified SMs and marked qualitative and quantitative variations among them. Treatment T16 showed the highest relative abundances for most shared phytoconstituents. Within this profile, compounds of high biological value stand out, such as *γ*-sitosterol (18.9%), followed by phytol acetate (8.14%), stigmasterol (6.39%), and phytol (5.04%). Significant percentages of *β*-sitosterol (2.12%), *α*-tocopherol (2.11%), campesterol (1.99%), *α*-tocospiro B (1.15%), and lupeol (0.71%) were also identified.

From a pharmacological perspective, identified SMs exhibit biological activities widely documented in the scientific literature. Within the tocopheroid group, *α*-tocospiro B is associated with antioxidant, antidiabetic, and cardioprotective effects [64,65,66]. *α*-tocopherol (vitamin E), for its part, is recognized for its potent antioxidant capacity and its role in modulating inflammatory and infectious processes; furthermore, its therapeutic potential has been investigated in leukemic and cardiovascular pathologies, as well as neurodegenerative disorders such as Alzheimer’s disease [67,68,69,70,71]. Regarding phytosterols, campesterol and stigmasterol exhibit a close structural and functional relationship, and are notable for their anti-inflammatory, cytotoxic, and antioxidant properties [72,73,74]. Similarly, *γ*-sitosterol and *β*-sitosterol have demonstrated remarkable efficacy in biological models as antidiabetic, antitumor, and anti-inflammatory agents [75,76,77]. Finally, identified terpenes exhibit a broad bioactive spectrum: lupeol, a pentacyclic triterpene, stands out for its versatility as a neuroprotective, hepatoprotective, and anticancer agent [78,79], while phytol, an acyclic diterpene, is primarily known for its antioxidant and antimicrobial properties [18].

In treatment T16, compounds with trace and minor concentrations were also identified, such as 13-tetradecenal (0.23%), oleic acid (0.28%), 1,8-nonadien-3-ol (0.06%), trans-geranylgeraniol (0.03%), betulin (0.02%), and the vitamin D derivative, 9,10-secocolesta-5,7,10(19)-triene-3,24,25-triol (0.01%); in addition to the significant presence of dihydroxanthine (1.21%) and *N*-aminopyrrolidine (5.26%) (Table 2). Among these, oleic acid, trans-geranylgeraniol, and betulin are of pharmacological interest given their proven antioxidant, anti-inflammatory, antimicrobial, and antitumor properties [80,81,82,83,84,85,86,87].

Taken together, these findings demonstrate that in vitro culture of *L. octovalvis*, under specific phytohormonal regulation schemes, allows for targeted modulation of metabolic biosynthetic routes, reducing phytochemical complexity while increasing the final yield of the SMs of interest. This strategy not only induces the biosynthesis of novel SMs in the species but also optimizes their accumulation, consolidating this biotechnological system as an efficient platform for obtaining bioactive precursors.

### 3.4. Antibacterial Activity

The minimum inhibitory concentration (MIC) values of gentamicin (positive control) and the hexane and ethyl acetate extracts obtained from *L. octovalvis* seedlings grown under basal (PW) and PGR-supplemented (T16) conditions are summarized in Table 3. In vitro antibacterial activity was evaluated against a panel of pathogens, including *Staphylococcus aureus*, methicillin-resistant *S. aureus* (MRSA), *Streptococcus pyogenes*, *Escherichia coli*, *Salmonella typhimurium*, and *Pseudomonas aeruginosa*.

Hexane extract from treatment T16 stood out, exhibiting the lowest MIC (125 µg/mL), surpassing the MIC of the MS group extracts (250 µg/mL). It is noteworthy that, against the MRSA strain, both hexane and ethyl acetate extracts of X7 exhibited the highest biological potency, with MICs < 62.5 µg/mL. In contrast, for *S. pyogenes*, the highest susceptibility (250 µg/mL) was recorded with the hexane extract of PW seedlings (Table 3). All extracts showed inhibitory activity against *S. aureus* and MRSA, highlighting the intrinsic antibacterial potential of *L. octovalvis.*

In contrast, Gram-negative bacteria showed limited susceptibility; *E. coli* and *S. typhimurium* exhibited resistance, with MICs > 1000 µg/mL across all treatments. For *P. aeruginosa*, only PW hexane extract exhibited an MIC of 250 µg/mL, whereas other extracts showed no significant inhibition (>1000 µg/mL). Activity observed against *S. aureus*, *E. coli*, and *P. aeruginosa* is consistent with reports for the wild plant [4,8,88]. Silva et al. [8,88] reported MIC value of 250 µg/mL for methanolic leaf extract of wild *L. octovalvis* against *S. aureus*, whereas Yakob et al. obtained values between 500 and 1000 µg/mL for leaf and stem extracts [4]. In the present work seedlings treated with RCV (T16) showed superior activity against *S. aureus* compared to reports from wild plants and basal in vitro culture; in addition, the activity observed against the clinically significant MRSA strain further highlights the therapeutic potential of these extracts. This potentiation of antibacterial activity could be attributed to the PGRs-induced biosynthesis of a broader repertoire of bioactive SMs in this specific treatment.

Fluctuations in MICs across different culture treatments are attributable to variations in their phytochemical profiles. Although both groups share phytoconstituents such as α-tocopherol, phytosterols (campesterol, stigmasterol, β-sitosterol), betulin, lupeol, and phytol, all with documented antimicrobial activity [89,90,91,92,93,94,95,96,97,98], differences in their relative concentrations and presence of exclusive SMs in T16 suggest the existence of synergistic effects that optimize the observed in vitro biological activity.

### 3.5. In Vitro Antioxidant Capacity

In vitro evaluation of the phytochemical potential and antioxidant capacity of *L. octovalvis* methanolic extracts reveals a clear difference in the concentration of secondary metabolites and redox efficiency between the analyzed fractions, designated extracts from plants without plant growth regulators (PW) and from plants under T16 treatment (T16) (Table 4).

Examining the phytochemical profile, extract T16 demonstrates a marked superiority in the biosynthesis or retention of polyphenolic compounds. This extract registers a total phenolic content of 59.57 ± 0.70 mg GAE/g, statistically significantly exceeding that of extract PW, which reports 44.02 ± 0.78 mg GAE/g. This homogeneous trend is observed in the quantification of total flavonoids, where the T16 fraction has twice the concentration of the PW fraction, with values of 8.53 ± 0.45 mg CAE/g and 4.56 ± 0.69 mg CAE/g, respectively.

The low proportion of flavonoids relative to the total phenolic content in both samples suggests that the chemical matrix of *L. octovalvis* is predominantly composed of other classes of phenolic acids or non-flavonoid polyphenols. This disparity in the density of phytoconstituents is directly proportional to the antioxidant bioactivity of the samples, measured by the Median Inhibitory Concentration (IC_50_), where a lower numerical value denotes greater scavenging power.

In the radical cation assay ABTS●+, the T16 extract exhibits a robust inhibitory capacity with an IC_50_ of 35.77 ± 0.39 µg/mL, positioning itself as a significantly more efficient reducing agent than the PW extract, whose value amounts to 98.62 ± 0.23 µg/mL. Although both extracts are kinetically distant from the pure molecular standard Trolox, which exhibits high affinity with an IC_50_ of 8.10 ± 0.20 µg/mL, fraction T16 maintains adequate competitive behavior within a complex crude matrix. Furthermore, scrutiny of antioxidant activity by hydrogen atom transfer to the stable radical DPPH validates the previous biological behavior.

Extract T16 again demonstrates greater efficacy with an IC_50_ of 59.47 ± 1.5 µg/mL, in contrast to extract PW, which requires almost twice concentration to achieve the same inhibitory effect, registering 104.35 ± 1.4 µg/mL. As expected for unpurified plant extracts, the two molecular complexes are located far from the action of gallic acid, an extremely potent polyhydroxylated positive control that has an IC_50_ of 1.75 ± 0.08 µg/mL.

It is metabolically consistent that the inhibition values for the DPPH radical are higher than those of ABTS in all the samples evaluated; this phenomenon is attributed to the kinetic restrictions imposed by the steric hindrance of the DPPH molecule and to the mixed nature of the electron transfer mechanisms of the ABTS system in hydrophilic phases [99,100,101].

In summary, the integrated data confirm the existence of a linear and inverse correlation between the phytochemical load and the IC_50_, demonstrating that the greater abundance of phenolic hydroxyl groups in extract T16 is the determining factor in the resonance stabilization of the evaluated root species.

In addition, DPPH and ABTS methods are sensitive for detecting antioxidant compounds, such as phenolic compounds, terpenes, terpenoids, and phytosterols [102,103]. In this regard, in vitro antioxidant capacity observed for treatment T16 could be associated with the presence of SMs such as phytol, oleic acid, α-tocopherol, stigmasterol, γ-sitosterol, and β-sitosterol, which were detected in ethyl acetate extract by GC-MS.

### 3.6. In Vivo Anti-Inflammatory Activity

Results of the TPA-induced ear edema model in male and female CD1 mice are presented in Table 5. The highest percentage of edema inhibition was observed with indomethacin (44.87% in males and 41.76% in females) at 2 mg/ear, while extract from treatment X7 (43.64% in males and 45.23% in females), showed statistical similar anti-edematous effect both applied at same quantity when compared to each TPA un-treated control (24.52 ± 0.99 mg in males, and 22.38 ± 0.64 mg in females); no statistically significant differences were found between these treatments in relation to edema formation per sex for each experimental group.

In male mice, a single dermal application of 0.5 mg/ear of T16 extract produced an effect comparable to that observed at 1 mg/ear (≈25%) for edema formation (≈17 mg). Compared with indomethacin, the anti-inflammatory effect of T16 extract at 0.5 and 1 mg/ear was statistically similar to that of 1 mg/ear indomethacin (22.93%); however, X7 treatment significantly achieved greater edema inhibition than indomethacin at 0.5 mg/ear.

In females, T16 treatment showed the same greater edema inhibition at 0.5 and 1 mg/ear (22.34 and 29.29%) as observed with indomethacin at the same quantity (9.12 and 19.71%, respectively).

Finally, it is noteworthy that the extract obtained from seedlings grown without PGRs T16 exhibited a significantly lower inhibitory effect at 2 mg/ear (23.97% in males and 20.17% in females), compared to both indomethacin and treatment at 2 mg/ear per sex.

Taken together, these results indicate that seedlings subjected to treatment T16 exhibit a notable anti-inflammatory effect in the TPA-induced ear edema model, even two-fold compared to those that grew in media not supplemented with PGRs (PW), moreover only those from treatment T16 generated a potency similar to the reference NSAID drug at the highest amount tested, an anti-edematous effect that was greater in both extracts (PW and T16) at the lowest amount tested compared to the drug that lost potency. This effect could be related to the phytochemical profile of ethyl acetate extract with greater phytochemical complexity and even new SMs, as determined by GC-MS, where those previously identified SMs have been reported to possess anti-inflammatory activity, including oleic acid, α-tocopherol, betulin, β-sitosterol, stigmasterol, phytol, and γ-sitosterol [83,104,105,106,107,108,109].

## 4. Conclusions

In vitro regeneration of *L. octovalvis* via indirect organogenesis is an efficient method not only for propagating the species but also for serving as a strategic biotechnological platform for controlled production of secondary metabolites of high biomedical value. Treatment T16, which combines BAP and NAA, established an optimal hormonal regimen for inducing shoot differentiation from callus, demonstrating that this morphogenic pathway is associated with plant’s phytochemical profile. Chemical characterization using GC-MS suggests that in vitro cultivation under controlled conditions modulates biosynthetic pathways that are not fully expressed in plants grown under basal (without PGRs) or wild conditions. This process resulted in the identification of 36 phytoconstituents, among which phytosterols, terpenes, and tocopherols stood out—compounds recognized for their biological relevance and their synergistic action in antioxidant and anti-inflammatory responses. These findings highlight the capacity of developing a biotechnological culture system to diversify and enhance the species’ metabolic repertoire. From a functional standpoint, extract obtained from T16 treated *L. octovalvis* cells showed superior performance compared to basal culture and previous reports of wild plants. In antibacterial assays, strong antibacterial activity was observed against clinically relevant pathogens, including methicillin-resistant *Staphylococcus aureus* (MIC < 62.5 µg/mL). Additionally, the extract exhibited moderate in vitro antioxidant capacity. Furthermore, in an in vivo model of ear edema, the extract showed significant anti-inflammatory activity, comparable to indomethacin at high doses and superior at low doses, with a consistent effect in both sexes. Taking together, these results establish a clear relationship between regeneration protocol, optimization of the phytochemical profile, and the increased biological potential of *L. octovalvis*. This study lays the foundation for using crops regenerated via indirect organogenesis as a sustainable source of bioactive compounds with promising applications in the development of phytopharmaceuticals.

## Figures and Tables

**Table 1:** Effect of plant growth regulators on the morphogenic response of *L. octovalvis* through different types of explants.

PGR	(mg/L)	Code	Leaf (%)	Node (%)
0	0	T0	0.00 ± 0.00 f	0.00 ± 0.00 e
NAA	0.1	T1	0.00 ± 0.00 f	0.00 ± 0.00 e
	0.5	T2	0.00 ± 0.00 f	25.00 ± 0.00 d
	1.0	T3	0.00 ± 0.00 f	0.00 ± 0.00 e
2, 4-D	0.1	T4	0.00 ± 0.00 f	0.00 ± 0.00 e
	0.5	T5	0.00 ± 0.00 f	0.00 ± 0.00 e
	1.0	T6	0.00 ± 0.00 f	0.00 ± 0.00 e
BAP	0.1	T7	0.00 ± 0.00 f	25.00 ± 0.00 d
	0.5	T8	25.00 ± 0.00 d	0.00 ± 0.00 e
	1.0	T9	0.00 ± 0.00 f	0.00 ± 0.00 e
KIN	0.1	T10	0.00 ± 0.00 f	30.00 ± 0.00 cd
	0.5	T12	0.00 ± 0.00 f	37.50 ± 12.50 bc
	1.0	T13	0.00 ± 0.00 f	30.00 ± 11.18 cd
BAP/NAA	0.1/0.1	T14	0.00 ± 0.00 f	0.00 ± 0.00 e
	0.1/0.5	T15	0.00 ± 0.00 f	0.00 ± 0.00 e
	0.1/1.0	T16	65.00 ± 0.00 b	0.00 ± 0.00 e
	0.5/0.1	T17	0.00 ± 0.00 f	0.00 ± 0.00 e
	0.5/0.5	T18	0.00 ± 0.00 f	0.00 ± 0.00 e
	0.5/1.0	T19	0.00 ± 0.00 f	0.00 ± 0.00 e
	1.0/0.1	T20	16.00 ± 7.83 e	0.00 ± 0.00 e
	1.0/0.5	T21	37.50 ± 12.50 c	0.00 ± 0.00 e
	1.0/1.0	T22	0.00 ± 0.00 f	0.00 ± 0.00 e
BAP/2, 4-D	0.1/0.1	T23	0.00 ± 0.00 f	0.00 ± 0.00 e
	0.1/0.5	T24	0.00 ± 0.00 f	0.00 ± 0.00 e
	0.1/1.0	T25	0.00 ± 0.00 f	0.00 ± 0.00 e
	0.5/0.1	T26	0.00 ± 0.00 f	0.00 ± 0.00 e
	0.5/0.5	T27	0.00 ± 0.00 f	0.00 ± 0.00 e
	0.5/1.0	T28	0.00 ± 0.00 f	0.00 ± 0.00 e
	1.0/0.1	T29	0.00 ± 0.00 f	0.00 ± 0.00 e
	1.0/0.5	T30	0.00 ± 0.00 f	0.00 ± 0.00 e
	1.0/1.0	T31	0.00 ± 0.00 f	0.00 ± 0.00 e
KIN/NAA	0.1/0.1	T32	0.00 ± 0.00 f	40.00 ± 0.00 b
	0.1/0.5	T34	0.00 ± 0.00 f	0.00 ± 0.00 e
	0.1/1.0	T35	25.00 ± 0.00 d	25.00 ± 0.00 d
	0.5/0.1	T36	0.00 ± 0.00 f	31.25 ± 10.83 cd
	0.5/0.5	T37	0.00 ± 0.00 f	25.00 ± 0.00 d
	0.5/1.0	T38	0.00 ± 0.00 f	30.00 ± 11.18 cd
	1.0/0.1	T39	58.33 ± 10.21 b	50.00 ± 0.00 a
	1.0/0.5	T40	83.33 ± 10.21 a	0.00 ± 0.00 e
	1.0/1.0	T41	0.00 ± 0.00 f	0.00 ± 0.00 e
KIN/2, 4-D	0.1/0.1	T42	0.00 ± 0.00 f	0.00 ± 0.00 e
	0.1/0.5	T43	0.00 ± 0.00 f	0.00 ± 0.00 e
	0.1/1.0	T44	0.00 ± 0.00 f	0.00 ± 0.00 e
	0.5/0.1	T45	0.00 ± 0.00 f	0.00 ± 0.00 e
	0.5/0.5	T46	0.00 ± 0.00 f	0.00 ± 0.00 e
	0.5/1.0	T47	0.00 ± 0.00 f	0.00 ± 0.00 e
	1.0/0.1	T48	33.33 ± 10.21 cd	0.00 ± 0.00 e
	1.0/0.5	T49	0.00 ± 0.00 f	0.00 ± 0.00 e
	1.0/1.0	T50	0.00 ± 0.00 f	0.00 ± 0.00 e

Dates are 30 days after inoculation. Mean ± standard deviation (SD) followed by the same superscript letter in the column are statistically similar at the *p* ≤ 0.05 level according to the Tukey multiple-range test. PGRs: Plant Growth Regulators. BAP: 6-Benzylaminopurine. KIN: Kinetin. NAA: Naphthaleneacetic acid. 2, 4-D: 2, 4-Dichlorophenoxyacetic acid.

**Table 2:** Compounds identified with GC-MS.

Compound	Extract	R_t_ (min)	PW	T13	T16	T32	T35	T36	T37	T40
1-Octanol, 2-butyl-	MeOH	6.617	-	-	-	-	-	-	-	15.95
	EtOAc	6.617, 22.71, 24.38, 27.433	43.06	-	-	1.85	-	1.17	-	-
endo-Borneol	EtOAc	9.304	-	-	-	-	-	-	-	1.71
α-Terpineol	EtOAc	9.777	-	-	-	-	-	-	-	1.82
*n*-hexadecanoic acid	Hex	18.874	-	0.33	-	3.64	0.25	-	-	-
(*Z*)-13-octadecenal	Hex	19.144	-	-	-	-	0.15	-	-	-
13-Tetradecenal	Hex	19.157	-	-	0.23	-	-	-	-	-
Oleic acid	Hex	20.536	-	-	-	2.07	-	-	0.79	-
	EtOAc	18.861, 27,453	-	-	0.28	-	-	-	-	-
Pterin-6-carboxylic acid	EtOAc	20.582	-	-	-	-	0.01	-	-	-
	MeOH	21.804, 26.507, 31.972	-	-	-	-	1.04	-	-	-
*trans*-Geranylgeraniol	EtOAc	28.629	-	-	0.03	-	-	-	-	-
	Hex	30.232	-	-	-	1.47	-	-	-	-
Squalene	Hex	28.642	8.26	18.47	-	9.48	4.54	14.26	7.79	4.92
α-tocospiro A	Hex	28.997	-	-	-	1.23	1.85	-	-	-
α-tocospiro B	Hex	29.04	-	-	1.15	0.83	-	0.28	1.1	1.97
Cucurbitacin b, 25-desacetoxy-	Hex	31.236	-	-	-	-	-	-	0.25	-
γ-tocopherol	Hex	31.42	0.28	0.55	-	-	0.63	-	1.14	6.66
Olean-12-ene-3,28-diol, (3β)-	Hex	31.48	-	-	-	-	0.33	-	-	-
9,10-Secocholesta-5,7,10(19)-triene-3,24,25-triol, (3β,5Z,7E)-	EtOAc	31.676	-	-	0.01	-	-	-	-	-
α-tocopherol	Hex	32.432	10.27	20.49	2.11	6.69	8.77	1.55	4.02	6.47
	EtOAc	32.432	-	-	0.1	0.03	0.02	-	-	-
Campesterol	Hex	33.897	1.52	1.71	1.99	2.11	2.18	2.44	16.4	1.14
Stigmasterol	Hex	34.33	1.92	3.78	6.39	4.82	5.06	15.23	3.25	2.78
	EtOAc	34.363	-	-	0.03	-	-	-	-	-
γ-sitosterol	EtOAc	35.329	-	-	0.16	-	-	-	-	-
	Hex	35.335	6.48	14.93	18.9	18.86	12.97	21.94	6.81	6.9
β-sitosterol	Hex	35.762	1.45	0.73	2.12	0.65	3.13	0.74	0.89	0.3
	EtOAc	36.774	-	-	0.01	0.05	0.04	-	-	-
Betulin	EtOAc	35.88	-	-	0.02	-	-	-	-	-
	Hex	37.963	0.54	-	-	-	-	-	-	0.09
Lupeol	Hex	36.701	0.12	0.67	0.71	0.56	0.26	1.36	1.34	0.37
Neophytadiene	EtOAc	12.227, 18.033	0.09	-	-	-	-	-	-	-
Dodecanoic acid, 3-hydroxy-	Hex	14.532, 20.49	0.67	-	-	-	-	-	-	-
1,8-Nonadien-3-ol	EtOAc	17.245, 20.582, 32.458	-	-	0.06	-	-	-	-	-
	MeOH	6.203, 7.537	-	-	-	-	-	-	-	7.03
Phytol, acetate	Hex	17.514, 20.654	8.9	3.78	8.14	7.86	4.68	6.91	3.52	4.39
	EtOAc	17.521, 22.723, 35.388	-	1.02	0.56	0.28	0.11	1.45	1.64	1.25
Phytol	Hex	17.613, 20.221	4.28	8.61	5.04	8.53	6.4	13.69	3.94	3.86
	EtOAc	20.221, 32.445	-	-	0.18	0.12	0.04	14.41	-	-
Dihydroxanthin	Hex	26.284, 31.906	0.13	0.45	1.21	-	-	0.81	0.51	0.8
Phytonadione	Hex	35.71, 42.574	-	-	-	-	-	-	-	0.71
2-Isopropylpiperazine	MeOH	6.125, 14.44	5.06	-	-	9.61	-	-	5.06	-
N-Aminopyrrolidine	MeOH	6.203, 14.44	-	-	5.26	-	-	-	-	-
5-Hydroxymethylfurfural	MeOH	7.537, 11.517	-	-	-	-	5.66	-	-	-
7-Hydroxy-3-(1,1-dimethylprop-2-enyl) coumarin	MeOH	8.22, 17.324, 21.889	-	-	-	2.24	-	-	-	-
Stearic acid hydrazide	MeOH	8.253, 11.517, 18.828	-	-	-	5.67	-	-	-	-

Relative abundance (%) of SMs. R_t_: retention time; PW: control without PGRs; -: not detected.

**Table 3:** Minimum inhibitory concentration of *L. octovalvis* seedlings’ extracts obtained by biotechnological culture.

Treatment	MIC (µg/mL)					
	*S. aureus* ATCC 6538	*MRSA* ATCC 4330	*S. p* ATCC 19615	*E. c* ATCC 8739	*S. typhi* ATCC 14028	*P. aeruginosa* Clinical Isolate
Gentamicin *	≤0.39	≤1.56	0.39	<0.39	<0.39	<0.39
PW-Hex	250	125	250	>1000	>1000	250
T16-Hex	125	<62.5	1000	>1000	>1000	>1000
PW- EtOAc	250	250	500	>1000	>1000	>1000
T16- EtOAc	250	<62.5	1000	>1000	>1000	>1000

PW-Hex: hexane extract of seedlings without PGR; T16-Hex: hexane extract of seedlings obtained with the T16 treatment; PW-EtOAc: ethyl acetate extract of seedlings without PGR; T16-EtOAc: ethyl acetate extract of seedlings obtained with the T16 treatment; *S. aureus*: *Staphylococcus aureus*; *MRSA*: *Methicillin-resistant Staphylococcus aureus*; *S. p*: *Streptococcus pyogenes*; *E. c*: *Escherichia coli*; *S. tiphy*: *Salmonella typhimurium*; *P. a*: *Pseudomonas aeruginosa*. * Reference compound (Sigma-Aldrich).

**Table 4:** Evaluation of the phytochemical potential and in vitro antioxidant capacity of *L. octovalvis* extracts.

Treatment	TPC (mg GAE/g Extract)	TFC (mg CAE/g Extract)	ABTS	DPPH
			IC_50_ (μg/mL)	
PW	44.02 ± 0.78 b	4.56 ± 0.69 b	98.62 ± 0.23 a	104.35 ± 1.4 a
T16	59.57 ± 0.70 a	8.53 ± 0.45 a	35.77 ± 0.39 b	59.47 ± 1.5 b
Trolox			8.10 ± 0.20 c	
Galic acid				1.75 ± 0.08 c

TPC: total phenolic content; TFC: total flavonoid content; PW: plants without PGR; T16: plants under treatment T16 (0.1 mg/L BAP + 1 mg/L NAA). Mean ± standard deviation (SD) followed by the same superscript letter in the column are statistically similar at the *p* ≤ 0.05 level according to the Tukey multiple-range test.

**Table 5:** Anti-inflammatory activity of ethyl acetate extracts from plantlets of *L. octovalvis* on ear edema development induced by TPA.

Treatment	Quantity (mg/ear)	Auricular Edema Formation (mg) (Edema Inhibition %)		Median Effective Quantity (EQ_50_, mg/ear)
		Males	Females	
TPA control	-	24.52 ± 0.99	22.38 ± 0.64	-
Indomethacin	0.5	21.64 ± 0.02^•*a^ (11.75%)	20.20 ± 0.25^•*a^ (9.12%)	Males: 4.07 Females: 4.07
	1	18.90 ± 0.34^•a^ (22.93%)	17.96 ± 0.38^•a^ (19.71%)	
	2	13.80 ± 0.65 ^a^ (44.87%)	13.03 ± 0.63 ^a^ (41.76%)	
PW	0.5	16.00 ± 0.57^•*ab^ (20.66%)	16.67 ± 0.56^•ab^ (12.28%)	Males: >5 Females: >5
	1	17.60 ± 0.41^•ab^ (12.72%)	16.00 ± 0.77 ^ab^ (15.78%)	
	2	15.33 ± 0.91 ^ab^ (23.97%)	15.16 ± 0.40 ^ab^ (20.17%)	
T16	0.5	17.92 ± 0.88^•ab^ (26.92%)	17.38 ± 0.36^•ab^ (22.34%)	Males: >2 Females: >2
	1	18.27 ± 0.87^•a^ (25.49%)	15.83 ± 0.69^•ab^ (29.29%)	
	2	13.82 ± 0.65 ^ac^ (43.64%)	12.26 ± 0.81 ^ac^ (42.23%)	

^*^ Each group represents the mean (±) with its SEM. Values in parentheses indicate the percent of ear edema inhibition with respect to the TPA control group, for each sex. Two-way ANOVA, post hoc Student Newman–Keuls (*p* ≤ 0.05), for each sex. • vs. 2 mg/ear. * vs. 1 mg/ear. a vs. TPA control. b vs. Indomethacin. c vs. PW. PGR: plant growth regulators. EQ_50_: median effective quantity. *n* = 6 for each experimental group per sex. PW: ethyl acetate extract from plants without plant growth regulators; T16: ethyl acetate extract from plants under T16 treatment.

## Data Availability

The data presented in this study is available on request from the corresponding author.
